# EphrinB2 Ameliorates Renal Fibrosis by Inhibiting the TGF-β/Smad3 Signaling Pathway and the Inflammation Response

**DOI:** 10.2174/0118715303366454250507042518

**Published:** 2025-05-12

**Authors:** Cheng Yuan, Qiuyuan Zhou, Feng Chen, Xueyun Gao, Ayinigaer Yusufu, Xiaoyan Wu, Lihua Ni

**Affiliations:** 1 Department of Oncology, Yichang Central People's Hospital and The First College of Clinical Medical Science, China Three Gorges University Yichang, Hubei, 443000 Yichang, China;; 2 Tumor Prevention and Treatment Center of Three Gorges University and Cancer Research Institute of Three Gorges University Yichang, Hubei, China;; 3 Department of Nephrology, Zhongnan Hospital of Wuhan University, Wuhan, Hubei 430071, China;; 4 Department of Pathology, the Central Hospital of Enshi Tujia and Miao Autonomous Prefecture, Enshi, 445000, China;; 5 Department of General Practice, Zhongnan Hospital of Wuhan University, Wuhan, Hubei, 430071, China

**Keywords:** Renal fibrosis, ephrinB2, TGF-β, unilateral ureteral obstruction, inflammation, chronic kidney disease (CKD)

## Abstract

**Background:**

EphrinB2 is known to play a variety of roles in the pathological process of fibrosis in the heart, skin, and retina, according to current research. However, the role of EphrinB2 in renal fibrosis remains to be clarified.

**Objective:**

We aimed to investigate the role of EphrinB2 in the renal fibrosis model and its underlying mechanisms.

**Materials and Methods:**

Unilateral ureteral obstruction (UUO) models and TGF-β-treated renal tubular epithelial cells (HK2) were adopted in this study to determine if EphrinB2 could lead to renal fibrosis.

**Results:**

EphrinB2 was highly expressed in renal tubular cells in UUO mice. Using adeno-associated virus (AAV)-mediated EphrinB2 overexpression, we observed significant improvements in renal function and injury, as well as a marked reduction in fibrosis. For example, EphrinB2 overexpression decreased the expression of fibrosis markers such as Fibronectin and α-SMA by approximately 40%. *In vitro*, EphrinB2 also significantly reduced extracellular matrix (ECM) deposition and cellular fibrosis under TGF-β stimulation. Mechanistically, EphrinB2 inhibited TGF-β/Smad3 signaling by approximately 40%, and reduced inflammatory markers such as MCP1 and IL-1β by approximately 60% and 35%, respectively.

**Dissusion and Conclusion:**

This study uncovered a previously unrecognized anti-fibrotic role of EphrinB2 in renal fibrosis, which is achieved through the prevention TGF-β/Smad3 signaling and inflammation response. It seemed that EphrinB2 might be a promising therapeutic target in the treatment of fibrotic diseases and kidney failure.

## 1 INTRODUCTION

Globally, chronic kidney disease (CKD) affects approximately 850 million individuals. Human health and society are at risk from this disease, which is still on the rise [1-4]. The presence of renal fibrosis is one of the most common pathological processes in CKD and contributes to end-stage renal disease (ESRD). Despite the development of several therapeutic strategies for renal fibrosis, such as empagliflozin [5, 6], Sirtuin3 activation strategies [7, 8], angiotensin converting enzyme inhibitors and Ang-II receptor blockers [9-12], mineralocorticoid antagonists [13-18] and so on, clinical treatments still only halt the progression of CKD, with kidney transplantation remaining the definitive treatment for ESRD. This highlights the urgent need for novel antifibrotic therapies to improve outcomes for patients with CKD and ESRD.

Renal fibrosis involves complex interactions between multiple cell types, both from the kidney and extrarenal sources. The progression of CKD is primarily driven by four types of cells: tubular epithelial cells (TECs), myofibroblasts, endothelial cells, and immune cells [19-22]. TECs, as key responders to renal injury, have garnered increasing attention in recent years, moving beyond the traditional focus on fibroblasts and myofibroblasts [23-29]. A growing number of recent studies are shedding light on the critical role of TECs in renal fibrosis [30-32]. When injured, TECs release paracrine signals, including pro-inflammatory and pro-fibrotic factors, into the renal interstitium, thereby influencing both inflammation and fibrogenesis [33]. These molecular signals contribute significantly to the progression of renal fibrosis and CKD, with a wide range of determinants identified in TECs that modulate fibrotic and inflammatory responses [34-36]. Understanding the molecular mechanisms underlying TEC involvement in renal fibrosis is crucial for developing targeted therapeutic strategies.

EphrinB2 (erythropoietin-producing hepatoma interactor B2) is a crucial bidirectional signaling molecule in mammals. Previously, we reported that EphrinB2 mediates high glucose-induced endothelial-to-mesenchymal transition in human aortic endothelial cells [37]. Recently, emerging evidences suggest EphrinB2 is implicated in fibrosis of multiple organs [38, 39]. Lagares *et al* reported that idiopathic pulmonary fibrosis patients have a higher level of EphrinB2, which induces human lung fibroblast differentiation into myofibroblasts [40]. While Kida *et al* indicated that activating EphrinB2 in mouse pericytes prevents myofibroblast activation to limit renal fibrosis after injury, suggesting that EphrinB2 plays an important protective role against kidney injury [41]. These findings suggest that EphrinB2 may have organ-specific roles in fibrotic diseases, where its signaling may either promote or inhibit fibrosis depending on the tissue type and cellular context. In the kidney, however, the role of EphrinB2 in TECs during fibrosis remains unclear. As TECs are central to initiating the tubulointerstitial fibrosis (TIF) response to injury, this study aims to investigate the role of EphrinB2 in TECs and elucidate the underlying mechanisms that contribute to renal fibrosis.

## 2 MATERIALS AND METHODS

### 2.1 Animal Models and Adeno-Associated Virus (AAV)

Thirty-two male C57BL/6 mice aged 6-8 weeks old (approximately 25 g body weight) were purchased from Hubei Beinert Biotechnology Co., Ltd. (Wuhan, China). The animal studies were under the Guide for the Care and Use of Laboratory Animals published by the National Institutes of Health. The present study was approved by the Animal Care and Ethics Committee of Wuhan University Zhongnan Hospital (ZN2023225).

The unilateral ureteral obstruction (UUO) model was used to induce renal fibrosis in mice, as previously described in our study [42]. This model is commonly employed to study tubulointerstitial fibrosis and is considered appropriate for investigating the pathophysiological mechanisms of renal injury in chronic kidney disease [43, 44]. To induce EphrinB2 overexpression in mice, we used adeno-associated virus (AAV) vectors to introduce EphrinB2 into the kidney *via* intrarenal injection. AAV containing empty plasmids (vehicle) was used as control. Four groups of mice were divided: (1) Sham + vehicle, (2) Sham + EphrinB2-OE, (3) UUO + vehicle, (4) UUO + EphrinB2-OE. Each group included eight mice. About 1×10^12^ genomic particles of AAV-csp-EphrinB2 (AAV-EphrinB2-OE) or AAV-csp-null (AAV-Ctrl-vehicle) from Hanbio Biotechnology (Shanghai, China) were delivered into the kidney by in situ kidney injection at six independent points. UUO was used to establish a renal interstitial fibrosis (RIF) model four weeks after intrarenal AAV injection.

### 2.2 Histological Examination and Assessment

Masson's trichrome, PAS staining, and hematoxylin and eosin (H&E) staining were performed on paraffin-embedded renal tissues from mice to evaluate histological changes and fibrosis. The renal tubule injury and collagen deposition were observed with an optical microscope (Olympus BX53, Japan). The lesions were quantified by ImageJ software (Image-Pro Plus 7.0).

### 2.3 Immunohistochemistry (IHC)

Our group followed the same process as our previous report [42].

To stain renal tissue sections for IHC, they were deparaffinized, hydrated, blocked with 3% H_2_O_2_, and incubated with specific primary antibodies overnight. Then they were incubated with a secondary antibody and DAB substrate. After counterstaining, dehydration in an ethanol gradient, and mounting with coverslips, the tissue sections were imaged using an optical microscope. The positive staining area was quantified using ImageJ software (Image-Pro Plus 7.0)

### 2.4 Transcriptomics Analysis

RNA quality was extracted and detected. The RNA concentration was detected by a NanoDrop 2000 spectrophotometer, and the quality and concentration of RNA samples were accurately and quantitatively detected by an Agilent 2100/4200. After the construction of the library, preliminary quantifications were conducted using Qubit 3.0, and RT-PCR was used for accurate quantification of the effective concentration of the library. Illumina NovaSeq 6000 was used for sequencing.

### 2.5 Cell Culture, Treatments, and Transfection

EphrinB2-OE lentivirus and its negative control virus were purchased from Genechem (Shanghai, China). According to the manufacturer's protocol, HK-2 cells were inoculated into a 6-well plate and infected with a lentivirus EphrinB2-OE or negative control virus, which was cultured at 37°C for 24 h. The cells were screened with purinomycin after passage, and the drug-resistant cells were used for subsequent tests [45].

Recombinant Human TGF-β1 (CHO derived, 100-21C) was obtained from Pepro Tech (US). The TGF-β1 intervention (10 ng/mL, 48 h) was applied to the EphrinB2-overexpressing stable cell line, and the cells were collected for further analysis. Using our previous report as a guide, the process was conducted [46].

### 2.6 Western Blotting

Our group followed the same process as our previous report [46, 47]. An overview of the antibodies is provided in Table **1**.

### 2.7 MG132 and CQ Treatment

To further investigate the mechanism of EphrinB2 downregulation under TGF-β treatment, HK2 cells were treated with chloroquine (CQ) and MG132. CQ, an autophagy inhibitor, was used at a concentration of 40 μmol/L for 6h to block autophagosome-lysosome fusion. MG132, a proteasome inhibitor, was applied at a concentration of 20 μmol/L for 6 h to inhibit proteasome activity. After the specified treatments, the cells were collected and analyzed for EphrinB2 protein levels to identify the pathway responsible for its degradation.

### 2.8 Statistical Analysis

We analyzed our data using GraphPad Prism 12 and performed unpaired two-tailed Student’s t-tests for comparisons between two groups. Statistical analyses were conducted by one-way ANOVA, followed by Bonferroni’s Multiple Comparison test. For statistical significance, a *P* value of 0.05 or less, or a *P* value of 0.01, was considered.

## 3 RESULTS

### 3.1 EphrinB2 Over-expression Improves Renal Injury in UUO Mice

**Fig. (1):**
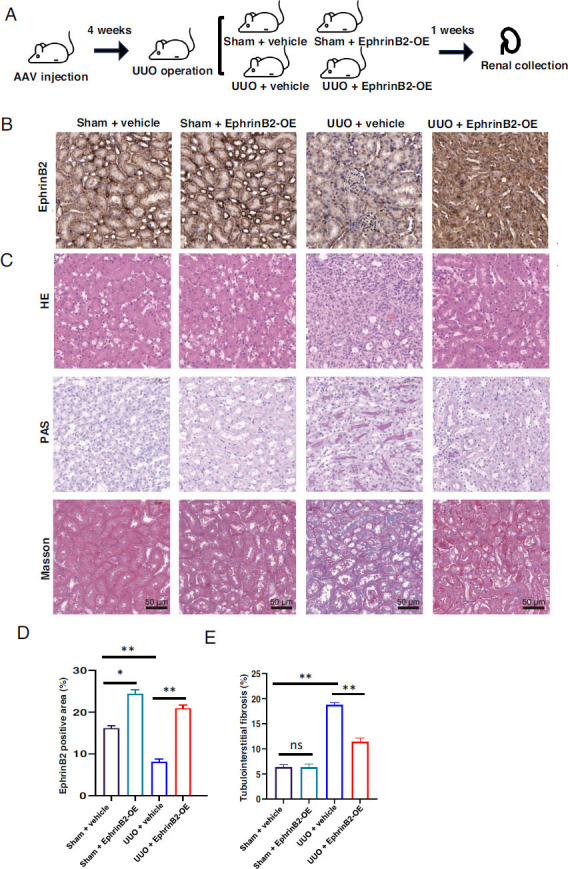
EphrinB2 overexpression improves renal injury in UUO mice. (**A**) Flowchart, (**B**) Immunohistochemistry staining detection for EphrinB2 in the kidney of mice, (**C**) Representative images of HE, PAS, and Masson staining (scale bar = 50 μm). (**D**) Representative images showing immunohistochemistry (IHC) staining of EphrinB2 (scale bar = 50 μm). (n = 3). (**E**) Tubulointerstitial fibrosis. (n = 8). The data are presented as the means ± SD. *p < 0.05, **p < 0.01, ns means no significance.

In research on CKD, UUO is commonly used as an animal model to investigate RIF mechanisms [48], which is characterized by inflammation and fibrosis. Then we evaluate the role of EphrinB2 *via* the UUO model (Fig. **1**). We used AAV-EphrinB2-OE transfection to induce the EphrinB2 overexpression into the mouse kidneys, followed by UUO surgery after 4 weeks (Fig. **1A**). EphrinB2 was successfully expressed in renal tubulointerstitial cells by AAV-mediated transduction (Figs. **1B** and **D**). H&E, PAS, and Masson staining were conducted to evaluate renal injury and fibrosis (Fig. **1C**). Based on H&E staining, EphrinB2 overexpression significantly diminished dilation of the renal tubular epithelium, interstitial edema, necrosis, and loss of the brush border. Similarly, PAS staining and Masson's trichrome staining revealed that UUO model mice overexpressing EphrinB2 had reduced collagen deposition and fibrosis in the tubulointerstitial area (Fig. **1E**). The findings indicate that EphrinB2 overexpression may enhance renal function and mitigate kidney injury in UUO model mice.

### 3.2 EphrinB2 Over-expression Improves Renal Fibrosis in UUO Mice

**Fig. (2):**
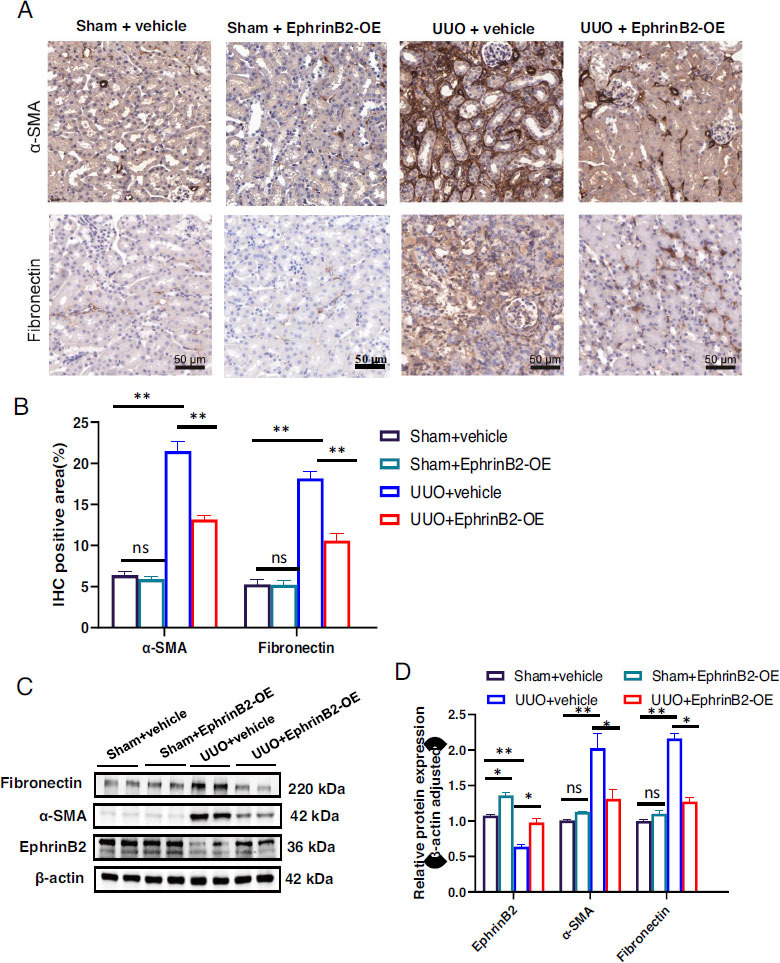
EphrinB2 overexpression improve renal fibrosis in UUO mice. (**A**) Representative images showing immunohistochemistry (IHC) staining of α-SMA and Fibronectin (scale bar = 50 μm). (**B**) Quantitative analysis of the IHC staining. (n = 3). (**C, D**) Protein expression levels (left) and relative quantitative data (right) of α-SMA and Fibronectin (n = 3). The data are presented as the means ± SD. *p < 0.05, **p < 0.01, ns means no significance.

An analysis of Fibronectin deposition (an ECM) was performed to determine how EphrinB2 affected renal fibrosis. In RIF, α-SMA plays a major role in promoting its progression. IHC staining revealed that the expression of Fibronectin, α-SMA in renal tissues was significantly decreased in UUO mice. EphrinB2 over-expression decreased the expression of Fibronectin and α-SMA compared to those in the model group (Figs. **2A** and **B**). The Western blot results are consistent with the IHC findings (Figs. **2C** and **D**). This notable reduction suggests that EphrinB2 overexpression may improve renal fibrosis in the UUO model mice.

### 3.3 EphrinB2 Over-expression Improves Renal Fibrosis *In Vitro*

**Fig. (3):**
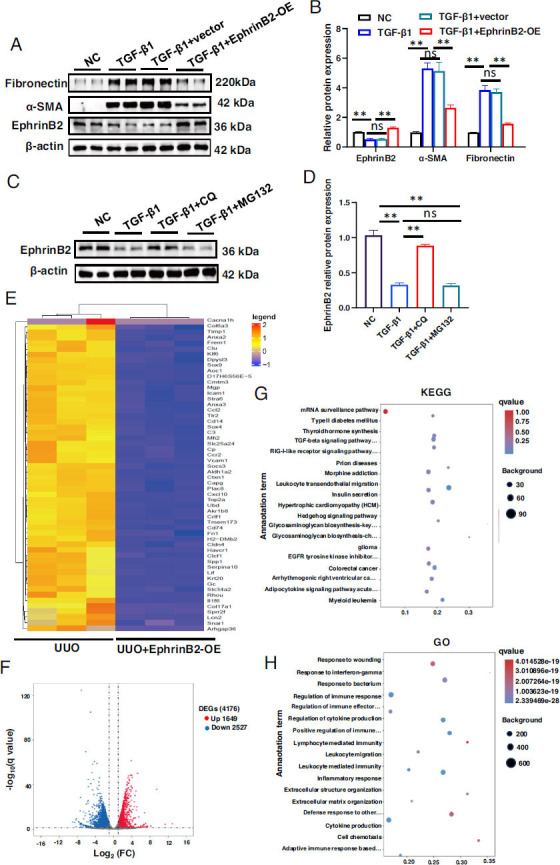
EphrinB2 overexpression improve renal fibrosis *in vitro*. (**A, B**) The protein expression levels (left) and relative quantitative data (right) of EphrinB2, α-SMA, and Fibronectin in HK2 cells with EphrinB2-OE infection (n = 3). (**C, D**) The protein expression levels (left) and relative quantitative data (right) of EphrinB2 in HK2 cells with CQ or MG132 treatment (n = 3). (**E, F**) Heatmap and volcano map showing differentially expressed genes (DEGs) in the UUO group and UUO+EphrinB2-OE group (n = 3). (**G, H**) KEGG enrichment and GO analysis of DEGs that were down-regulated by EphrinB2 over-expression. The data are presented as the means ± SD. **p > 0.01, ns means no significance.

To further determine the role of EphrinB2 in renal fibrosis progression, we overexpressed EphrinB2 in HK2 cells. As depicted in Figs. (**3A** and **B**), we demonstrated that TGF-β-elicited increases of Fibronectin and α-SMA were further decreased in EphrinB2-up-regulated HK2 cells. Importantly, we found that TGF-β treatment could decrease the EphrinB2 protein expression in HK2 cells. Ubiquitination-proteasome system (UPS) and autophagy are two main protein degradation machineries. We then sought to make clear whether the degradation of EphrinB2 was mediated by the proteasome or autophagy. The autophagy inhibitor chloroquine (CQ) and the proteasome inhibitor (MG132) were used in TGF-β1-treated HK2 cells to identify how EphrinB2 was reduced. We found that CQ blocked the loss of EphrinB2 while MG132 made no change (Figs. **3C** and **D**). These data indicated that TGF-β induced EphrinB2 degradation through the autophagy-lysosome pathway.

### 3.4 Transcriptomics Analysis Reveals the Mechanisms behind EphrinB2's Ability to Alleviate Renal Fibrosis

Considering that EphrinB2 exerts a significant anti-fibrotic effect both *in vitro* and *in vivo*, we plan to further study the underlying mechanisms through RNA-sequence analysis. Separation of the UUO group and the UUO+EphrinB2-OE group can be seen on a heatmap (Fig. **3E**). As compared to the UUO group, the UUO+ EphrinB2-OE group had 4176 DEGs in comparison to the UUO group. Upregulation affected 1649 DEGs, and downregulation affected 2527 (Fig. **3F**).

An enrichment analysis based on transcriptomics was conducted in order to gain further insight into the mechanisms involved. The KEGG and GO analyses revealed a significant association between TGF-β signalling pathway as well as inflammation response between the UUO group and UUO+ EphrinB2-OE group (Figs. **3G** and **H**).

**Fig. (4):**
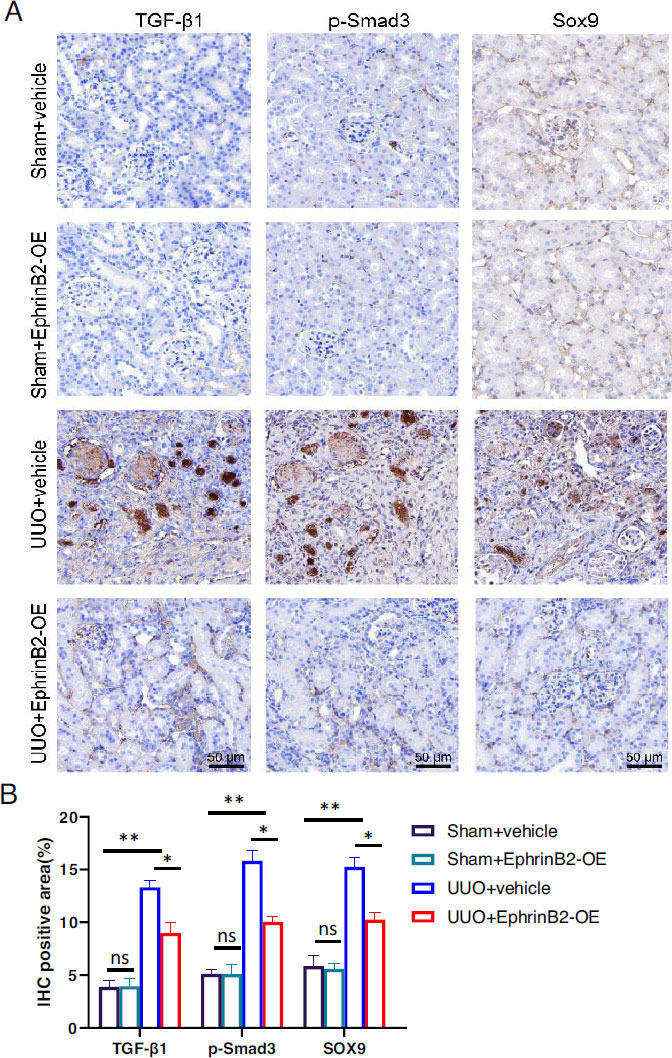
EphrinB2 overexpression alleviated the TGF-β pathway. (**A**) Representative images showing IHC staining of TGF-β1, p-Smad3 and Sox9 (scale bar = 50 μm), and (**B**) Relative quantitative data. The data are presented as the means ± SD. (n=3). *p < 0.05, **p < 0.01, ns means no significance.

**Fig. (5):**
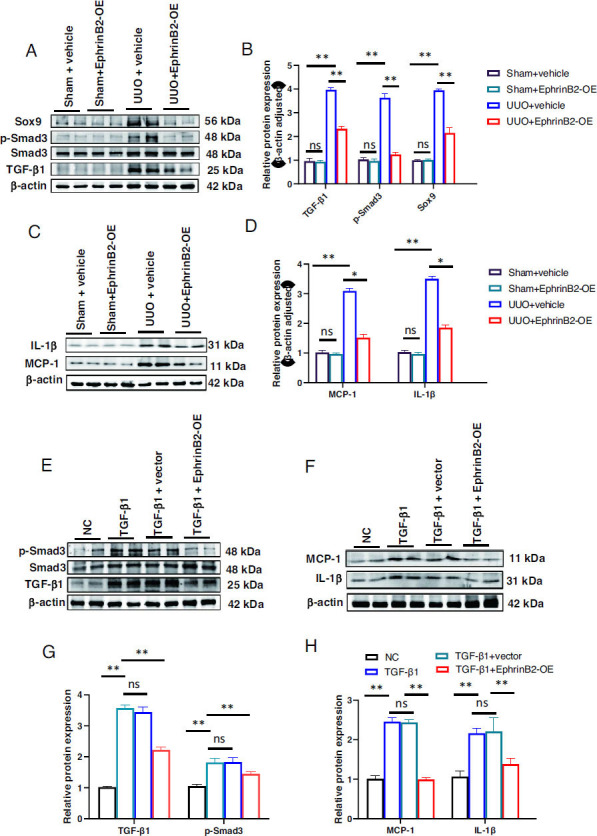
EphrinB2 overexpression alleviated the inflammation response (**A, B**) The protein expression levels (left) and relative quantitative data (right) of Sox9, p-Smad3, Smad3, and TGF-β1 in mouse renal cells with or without EphrinB2 overexpression (n = 3). (**C, D**) The protein expression levels (left) and relative quantitative data (right) of MCP-1 and IL-β1 in mouse renal cells with or without EphrinB2 over-expression (n = 3). (**E, G**) The protein expression levels (upper panel) and relative quantitative data (lower panel) of p-Smad3, Smad3, and TGF-β1 in HK2 cells with or without EphrinB2 overexpression (n = 3). (**F, H**) The protein expression levels (upper panel) and relative quantitative data (lower panel) of MCP-1 and IL-1β in HK2 cells with or without EphrinB2 over-expression (n = 3). The data are presented as the means ± SD. *p < 0.05, **p < 0.01, ns means no significance.

According to transcriptomics, the immunohistochemistry (IHC) and substantial upregulation of proteins involved in TGF-β1 signalling (TGF-β1, p-Smad3, Sox9) in response to UUO. Importantly, EphrinB2 over-expression prevented their overexpression (Figs. **4A**, **B** and **5A**, **B**). Moreover, in the UUO group, there was enhanced inflammation response (IL-1β and MCP-1), and EphrinB2 over-expression reversed this increase (Figs. **5C** and **D**).

To further confirm the *in vivo* findings above, we then examined the TGF-β/Smad pathway and inflammation response-related proteins *in vitro*. A significant increase in TGF-β1, p-Smad3, as well as MCP-1 and IL-1β expressions was discovered in HK2 cells after TGF-β1 incubation. In addition, EphrinB2 overexpression attenuated TGF-β1, p-Smad3, as well as MCP-1 and IL-1β expressions (Figs. **5E**-**H**).

These data indicated that EphrinB2 alleviated TGF-β signalling pathway and inflammation response, which might be associated with the anti-fibrotic effects.

## 4 DISCUSSION

RIF is the major pathophysiology associated with almost all CKD cases [49]. Several studies [50-55], including ours [46], have evaluated the cellular and molecular mechanisms underlying renal fibrosis. However, existing treatments primarily focus on preventing further kidney damage and controlling risk factors, but they do not directly address the underlying mechanisms of fibrosis. In our study, we uncovered a previously unrecognized anti-fibrotic role of EphrinB2 in renal fibrosis, which is achieved through preventing TGF-β/Smad signaling and inflammation response. Our findings highlight EphrinB2 as a promising therapeutic target for managing fibrotic kidney diseases, particularly in cases where current therapies offer limited efficacy.

Despite EphrinB2's role in angiogenesis and development [56-58], it remains unclear how it contributes to organ fibrosis. In skin, cardiac, and lung, EphrinB2 was identified as a promoter of fibrogenesis [40, 59, 60]. Kida *et al* indicated that EphrinB2 signaling in mouse pericytes inhibits their transition to myofibroblasts, thereby preventing renal fibrosis [41]. Tubular cells are the initiator of the TIF response to a variety of injuries. Thus far, the role of tubular EphrinB2 in renal fibrosis remains largely unclear. Our *in vivo* and *in vitro* data supported that EphrinB2 inhibits the TGF-β/Smad3 pathway, preventing renal fibrosis. Additionally, it reduces pro-inflammatory cytokines like MCP-1 and IL-1β, which play a role in immune cell recruitment and fibrosis progression. The diverse functions of EphrinB2 across various tissues can be attributed to multiple factors. Firstly, the cellular and molecular environments differ markedly among organs, thereby influencing the interaction of EphrinB2 with downstream signaling pathways. Secondly, the presence or absence of specific co-factors, receptors, and other molecular mediators can modify the role of EphrinB2. Thirdly, the mode of EphrinB2 expression-whether transient, sustained, or at varying levels-may result in differing outcomes in fibrosis. Lastly, the nature and severity of the injury or disease model under investigation can also determine the functional role of ephrin B2 in a specific tissue. In our study, we demonstrated a new mechanism of EphrinB2 inhibition of renal fibrosis by lowering the signaling of TGF-β/Smad3 and inflammation response.

TGF-β signaling has been demonstrated to play central roles in mediating renal fibrosis, and induces renal scarring largely by activating its downstream Smad3 signaling pathway [61-66]. Phosphorylated Smad proteins form complexes that translocate to the nucleus, where they regulate the expression of genes involved in fibrosis [67, 68]. This process promotes the activation of fibroblasts, increased ECM production, and the deposition of collagen, ultimately leading to the progression of renal fibrosis [61, 69]. Present results, therefore, support the notion that TGF-β/Smad3-related proteins TGF-β and p-Smad3 are increased in renal fibrosis models compared to controls. And EphrinB2 overexpression in UUO mice and HK2 cells suppressed the levels of TGF-β/Smad3 signaling pathway- and targeted- related proteins, suggesting that EphrinB2 may mitigate renal damage by inhibiting the TGF-β/Smad3 signaling pathway and the downstream targeted pro-fibrotic proteins during renal fibrosis.

Apart from TGF-β/Smad3 signalling, chronic and persistent inflammation is a key driver in the progression of renal fibrosis [3, 28, 70-76]. Upon injury, renal TECs are activated and release a variety of pro-inflammatory cytokines and chemokines, which play a crucial role in the recruitment of immune cells and the activation of fibroblasts [77, 78]. This inflammatory cascade contributes significantly to the fibrotic remodeling of the kidney tissue. The results of this study show that fibrotic kidneys have increased levels of inflammatory cytokines (such as IL-1β and MCP-1), and EphrinB2 overexpression could downregulate these expressions. These results underscore the potential of EphrinB2 as a therapeutic target that could not only inhibit fibrosis but also alleviate the inflammatory component of renal injury, offering a dual approach to the treatment of kidney fibrosis. With current treatments often inadequate, EphrinB2 offers a novel approach that could enhance existing therapies, potentially improving outcomes for patients with advanced renal fibrosis.

There are some limitations to our study. Our data demonstrated that TGF-β/Smad3 signaling and inflammation response were reduced after EphrinB2 overexpression during renal fibrosis. However, a deeper understanding of EphrinB2’s relationship with the two pathways, as well as how EphrinB2 regulates the pathways, is still needed. Additionally, the UUO model, though insightful, may not fully mimic human CKD, so future studies should use more clinically relevant models or patient samples. Lastly, we focused on short-term outcomes, and long-term studies are necessary to evaluate the durability of these effects and any potential adverse outcomes.

## CONCLUSION

In summary, our study identifies a previously unrecognized anti-fibrotic role of EphrinB2 in renal fibrosis. Using both *in vivo* and *in vitro* models, we demonstrate that overexpression of EphrinB2 in TECs alleviates renal injury, reduces ECM deposition, and mitigates cellular fibrosis. Mechanistically, these effects are associated with the suppression of TGF-β/Smad3 signaling and the attenuation of inflammatory responses. These findings suggest that targeting EphrinB2 may provide a novel therapeutic approach for the treatment of fibrotic kidney diseases.

## Figures and Tables

**Table 1:** The information about the antibodies.

Antibody	Species	Company	Article No.
EphrinB2	Mouse	Santa	SC-398735
α-SMA	Rabbit	CST	19245
Fibronectin	Rabbit	Proteintech	15613-1-AP
Smad3	Rabbit	ABclonal	A16913
Phospho-Smad3	Rabbit	ABclonal	AP0727
Sox9	Rabbit	Baijia	IPB0609
TGF-β1	Mouse	Santa	SC-52893
IL-1β	Rabbit	ABclonal	A23484
MCP-1	Mouse	Santa	SC-52701
β-actin	Mouse	Proteintech	66009

## Data Availability

The data of this study are available upon request by contacting the corresponding author.

## LIST OF ABBREVIATIONS

CKD: Chronic Kidney Disease
UUO: Unilateral Ureteral Obstruction
ECM: Extracellular Matrix
ESRD: End-Stage Renal Disease
TECs: tubular epithelial cells
AAV: Adeno-Associated Virus
RIF: Renal Interstitial Fibrosis
H&E: Hematoxylin and Eosin
